# Assessing the efficacy of two dual-active ingredients long-lasting insecticidal nets for the control of malaria transmitted by pyrethroid-resistant vectors in Benin: study protocol for a three-arm, single-blinded, parallel, cluster-randomized controlled trial

**DOI:** 10.1186/s12879-021-05879-1

**Published:** 2021-02-19

**Authors:** Manfred Accrombessi, Jackie Cook, Corine Ngufor, Arthur Sovi, Edouard Dangbenon, Boulais Yovogan, Hilaire Akpovi, Aurore Hounto, Charles Thickstun, Gil G. Padonou, Filemon Tokponnon, Louisa A. Messenger, Immo Kleinschmidt, Mark Rowland, Martin C. Akogbeto, Natacha Protopopoff

**Affiliations:** 1 Faculty of Infectious and Tropical Diseases, Disease Control Department, London School of Hygiene and Tropical Medicine, WC1E 7HT, London, UK; 2 Medical Research Council (MRC) International Statistics and Epidemiology Epidemiology Group, London School of Hygiene and Tropical Medicine, WC1E 7HT, London, UK; 3 Centre de Recherche Entomologique de Cotonou (CREC), Cotonou, Benin; 4 Faculty of Agronomy, University of Parakou, Parakou, Benin; 5 National Malaria Control Program, Ministry of Health, Cotonou, Benin; 6 School of Epidemiology and Public Health, Faculty of Medicine, University of Ottawa, Ottawa, Canada; 7 School of Pathology, Faculty of Health Sciences, University of Witwatersrand, Johannesburg, South Africa; 8 Southern African Development Community Malaria Elimination Eight Secretariat, Windhoek, Namibia

**Keywords:** Dual-active ingredient long-lasting insecticidal nets, Chlorfenapyr, pyriproxyfen, Royal Guard®, Interceptor® G2, Malaria case incidence, Malaria prevalence, Entomological inoculation rate, Cluster randomized controlled trial, Benin

## Abstract

**Background:**

Long-lasting insecticidal nets (LLINs) are currently the primary method of malaria control in sub-Saharan Africa and have contributed to a significant reduction in malaria burden over the past 15 years. However, this progress is threatened by the wide-scale selection of insecticide-resistant malaria vectors. It is, therefore, important to accelerate the generation of evidence for new classes of LLINs.

**Methods:**

This protocol presents a three-arm superiority, single-blinded, cluster randomized controlled trial to evaluate the impact of 2 novel dual-active ingredient LLINs on epidemiological and entomological outcomes in Benin, a malaria-endemic area with highly pyrethroid-resistant vector populations. The study arms consist of (i) Royal Guard® LLIN, a net combining a pyrethroid (alpha-cypermethrin) plus an insect growth regulator (pyriproxyfen), which in the adult female is known to disrupt reproduction and egg fertility; (ii) Interceptor G2® LLIN, a net incorporating two adulticides (alpha-cypermethrin and chlorfenapyr) with different modes of action; and (iii) the control arm, Interceptor® LLIN, a pyrethroid (alpha-cypermethrin) only LLIN. In all arms, one net for every 2 people will be distributed to each household. Sixty clusters were identified and randomised 1:1:1 to each study arm. The primary outcome is malaria case incidence measured over 24 months through active case detection in a cohort of 25 children aged 6 months to 10 years, randomly selected from each cluster. Secondary outcomes include 1) malaria infection prevalence (all ages) and prevalence of moderate to severe anaemia in children under 5 years old, measured at 6 and 18 months post-intervention; 2) entomological indices measured every 3 months using human landing catches over 24 months. Insecticide resistance intensity will also be monitored over the study period.

**Discussion:**

This study is the second cluster randomised controlled trial to evaluate the efficacy of these next-generation LLINs to control malaria transmitted by insecticide-resistant mosquitoes. The results of this study will form part of the WHO evidence-based review to support potential public health recommendations of these nets and shape malaria control strategies of sub-Saharan Africa for the next decade.

**Trial registration:**

ClinicalTrials.gov, NCT03931473, registered on 30 April 2019.

**Supplementary Information:**

The online version contains supplementary material available at 10.1186/s12879-021-05879-1.

## Background

Insecticide-treated nets (ITNs) and, more recently, long-lasting insecticidal nets (LLINs) are the most widely used preventive measure for controlling malaria in sub-Saharan Africa (SSA) [1]. The World Health Organization (WHO) estimates that over 50% of the SSA population now sleep under LLINs and together with improved diagnosis and treatment, nets have contributed to an estimated 42 and 66% reduction in malaria incidence and mortality, respectively, over the last 15 years [2]. Pyrethroids are currently the only type of insecticide used routinely on LLINs, and the rapid spread of pyrethroid-resistant vectors seriously threatens to reverse the gains achieved so far [3]. Indeed, several studies have demonstrated that LLINs are becoming less effective at killing mosquitoes in areas of high resistance compared to areas of susceptibility [4, 5], although epidemiological evidence remains inconclusive in some studies [6, 7].

Due to concerns about the potential failure of current control tools as a result of insecticide resistance, WHO has encouraged manufacturers to develop new types of LLINs as part of the Global Plan for Insecticide Resistance Management in malaria vectors (GPRIM) [8]. The first nets to contain a mixture of active ingredients with evidence for impact on epidemiological outcomes were nets that combined a pyrethroid insecticide with the synergist piperonyl butoxide (PBO) which restores susceptibility to pyrethroid by neutralising mixed-function oxidase function responsible for resistance in vectors [9, 10].

New classes of ITNs combining two insecticides with differing modes of action could have the potential to improve vector control and delay the evolution of resistance and preserve the lifespan of both active ingredients (AI). The two most advanced products are the pyrethroid-pyriproxyfen LLIN (Olyset® Duo and Royal Guard®) [11–14] and a pyrethroid-chlorfenapyr LLIN (Interceptor® G2) [15–17]. Both types of LLINs have demonstrated improved efficacy in entomological studies but currently, limited epidemiological evidence has been generated. Most of the available evidence for these new nets are based on Phase I laboratory studies and Phase II experimental hut trials. Only Olyset® Duo has been tested in an randomized clinical trial (RCT) in Burkina Faso and demonstrated efficacy on malaria case reduction in comparison to clusters using standard pyrethroid nets [12].

There are currently no published data on the impact of Royal Guard® and Interceptor® G2 on malaria epidemiological outcomes. These LLINs need to be evaluated in the community to determine the full extent of their effectiveness for malaria control. Failure to do so will – as shown by the recent history of PBO LLINs [18, 19] – delay the scale-up of new and potentially effective tools due to lack of malaria control evidence. To receive a public health recommendation from WHO, new types of LLIN need to demonstrate improved epidemiological efficacy compared to standard pyrethroid LLIN in two settings [20]. A trial evaluating the same dual-AI LLINs is currently ongoing in Tanzania (clinical.gov, NCT03554616). West Africa constitutes a different environment and ecology from East Africa, with historically higher intensity of pyrethroid resistance in malaria vectors [21] and so far there is only limited entomological and epidemiological evidence that new mixture nets would have a better effect on malaria indicators compared to standard nets [14, 15].

The main objective of the present study is to assess the efficacy of two types of dual-AI LLINs on malaria in a setting where the main malaria vectors are resistant to pyrethroid insecticides. Secondary objectives are to assess the durability and bio-efficacy of the dual-AI LLINs in the community [22]; and to examine whether entomological outcomes from adapted experimental hut trials can predict epidemiological and transmission outcomes of the RCT using a malaria transmission model developed by Churcher et al. [23, 24]. The study protocol is reported in line with the Standard Protocol Items: Recommendations for Interventional Trials (SPIRIT) 2013 Statement [25].

## Study objectives

### Epidemiology

#### Primary objective

To evaluate the efficacy of two types of dual-AI LLINs compared to standard pyrethroid LLINs on malaria case incidence in children aged 6 months to 10 years during two years of follow up.

#### Secondary objectives

- To assess the efficacy of each dual-AI LLIN compared to a standard pyrethroid-only LLIN on malaria infection prevalence in all age groups, and prevalence of moderate to severe anaemia in children under 5 years old, at 6 and 18 months post-net distribution.
- To assess if dual-AI LLINs have a safety profile similar to pyrethroid-only LLINs in the population of the trial study area.
- To monitor and evaluate the equity of study nets coverage and usage in the trial.

### Entomology

#### Primary objective

To assess the efficacy of each dual-AI LLINs compared to a standard pyrethroid-only LLIN on mosquito density, sporozoite rate, and the entomological inoculation rate (EIR) as a measure for malaria transmission.

#### Secondary objectives

- To assess the impact of the new type of LLINs on *Anopheles* vector survivorship and other entomological outcomes (e.g., mosquito resting behaviour, species composition, feeding, ovary development, and fecundity).
- To assess annual changes in insecticide resistance intensity to the three insecticides used on the nets.

## Methods/design

### Study area

**Fig. 1:**
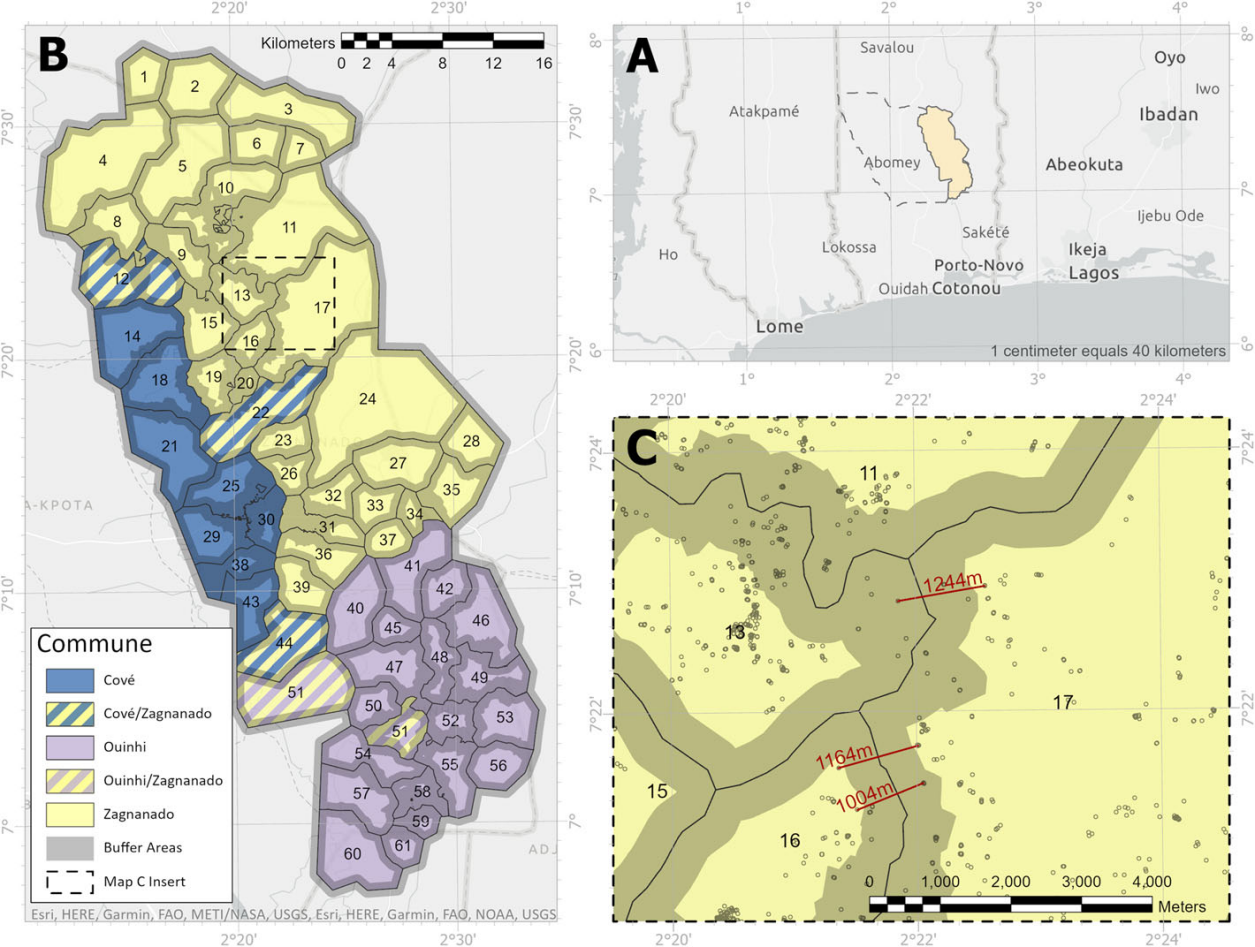
Study area. Map showing Cove, Zagnanado and Ouinhi Districts, located in Zou department, central Benin, West Africa (Panel **a**); the 60 study clusters identified with core and buffer area and intervention allocation (Panel **b**); a minimum of 1000 m area was created between households in adjacent clusters (Panel **c**). Source of map: Own from the study investigators (CT, JC, MA, ED).

The study site is situated in Cove, Zagnanado, and Ouinhi Districts, located in the Zou department, central Benin, 154 km north of Cotonou, the economic capital (Fig. 1). The area was selected because of (i) a prevalence of malaria infection between 20 to 40%, (ii) high pyrethroid resistance in the main malaria vectors, (iii) and proximity to experimental hut sites. This area consists of 123 villages with approximately 54,000 households and population size of 220,000 inhabitants. The main economic activities of the population are farming, fishing, hunting, and trading [26, 27].

Malaria is highly endemic, and transmission occurs year-round. There are two rainy seasons, from April – July and from October – November. Malaria infection prevalence in the Zou department was 20% in children under 5 years old according to the demographic health survey (DHS) conducted in 2011–2012 [28] and increased to 36.5% in the 2017–2018 DHS [29]. The main vector control provision in the Zou department consists of the distribution of pyrethroid-only LLINs through regular universal coverage campaigns (most recently in 2017) and routine service delivery to pregnant women during antenatal care and to children under 5 years during extended programmes on immunization. According to the 2018 DHS, ITN usage in children under 5 years was 78.2%.

The main vector species are *Anopheles coluzzii* and *Anopheles gambiae* sensu stricto. Entomological surveys performed in the Cove region in 2015 revealed high levels of pyrethroid resistance intensity (> 200 fold) mainly due to high frequencies of kdr of > 90% and elevated cytochrome P450 enzymes [30].

### Trial design

**Fig. 2:**
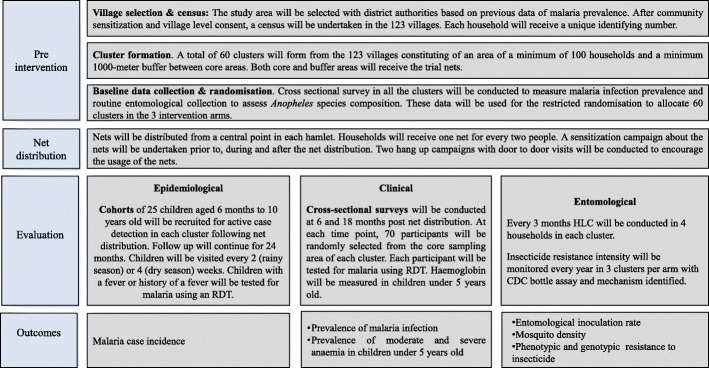
Summary of study design

The trial is a three-arm superiority, single-blinded, cluster-randomized trial with 20 clusters in each arm (60 clusters in total). The 3 arms are (i) mixture pyrethroid and chlorfenapyr LLIN: Interceptor® G2 (Intervention 1), (ii) mixture pyrethroid-pyriproxyfen LLIN: Royal Guard® (Intervention 2), (iii) pyrethroid-only standard LLIN: Interceptor® (Control/reference arm). The study design is summarized in Fig. 2.

Each cluster will be comprised of 1 village or a group of villages for an average of 200 households (1200 residents) per cluster. Clusters will have a minimum of 100 children and 100 households. A “fried egg” design will be used for cluster boundaries. Clusters will be designed with core and buffer areas to reduce the likelihood of spill-over of intervention effects. Nets will be distributed to all households in a cluster (i.e. core and buffer areas) but data collection to measure intervention effects will be restricted to households situated in the core areas to avoid spillover from neighbouring clusters receiving different nets. The cluster demarcation will be performed using the spatial analyst toolbox in ArcGIS (ESRI, Redlands, USA) based on the following criteria; a minimum of 50 children aged 6 months to 10 years in the core area and a buffer of minimum 1000 m between core households and any households in an adjacent cluster.

To estimate malaria case incidence, a cohort of 25 children per cluster aged 6 months to 9 years will be randomly selected from a census list of children living in the study clusters and followed over 24 months. For malaria infection prevalence assessment, two cross-sectional surveys will be conducted at 6 and 18 months post-net distribution; 70 individuals per cluster will be randomly selected for each cross-sectional survey, stratified by age (< 5 years, 5–9 years, 10–14 years, ≥15 years and older) from each of the 60 clusters.

Routine cross-sectional entomological surveys using human landing catches (HLCs) will be conducted in all clusters to assess the human biting rate and EIR. Each cluster will be visited once every three months. Four households will be selected from a census list in each study cluster at each time point. The first house will be randomly selected by the study data manager, while the other three will be chosen by the field team, in a radius of 15–20 m around the first one, to facilitate the supervision of mosquito collectors.

### Eligibility criteria

The cohort will include children aged 6 months to 9 years old resident in the study villages, without any severe illnesses and whose parents/carers have given written informed consent for their child to be included in the study. Inclusion criteria during cross-sectional surveys will be the willingness to participate and provide consent (or for parents/carers to provide consent for children); resident in the village during the previous 3 months, selected individuals without severe illness.

### Interventions

#### Description of the study nets

The nets are blue, rectangular, and identical sizes (1.8 m long, 1.6 m wide, and 1.8 m high); however, the Royal Guard® textile is different from the other two nets (details below). The three net brands are all WHO pre-qualified vector control products [31].

Intervention 1: Interceptor® G2 (BASF Corporation) is a mixture LLIN made of polyester netting (100 deniers) coated with a wash-resistant formulation of 200 mg/m^2^ chlorfenapyr and 100 mg/m^2^ alpha-cypermethrin. Chlorfenapyr is a member of the pyrrole class of insecticides [32] and disrupts cellular respiration and oxidative phosphorylation in mitochondria and due to this unique mode of action is toxic to mosquitoes that are resistant to standard neurotoxic insecticides like pyrethroids [33]. No record of cross-resistance to chlorfenapyr has been reported up to now.

Intervention 2: Royal Guard® (Disease Control Technologies, LLC) is a mixture LLIN made of polyethylene (120 deniers) incorporating 225 mg/m^2^ pyriproxyfen and 261 mg/m^2^ alpha-cypermethrin. Pyriproxyfen is a multiple acting compound (known as a juvenile hormone analogue) with insecticidal and sterilising effects on females which is known to disrupt reproduction and fertility of eggs [34].

Control: Interceptor® LLIN (BASF Corporation) is a pyrethroid-treated LLIN with alpha-cypermethrin (coated onto filaments) at a target dose of 200 mg/m^2^ of polyester fabric (100 deniers). Interceptor was chosen as a direct comparison to Interceptor G2 and Royal Guard as all three nets are impregnated with alpha-cypermethrin pyrethroid (albeit at different concentrations) and because some pyrethroid resistance mechanisms involving CYP6 genes are specific to type I (permethrin) or type II (alpha-cypermethrin) pyrethroids [35].

#### Intervention description

Nets will be distributed with the support of the National Malaria Control Programme (NMCP). All households in the study area will receive one net for every two people. The census listing will be used to facilitate net distribution at a central location in each hamlet. To reach a minimum of 85% access following the distribution and a minimum of 75% usage, information, education, and communication (IEC) activities will be conducted before, during, and after LLIN distribution to increase usage in the study area, including instructions on when and how to wash the nets. A door to door hang-up campaign will take place after the distribution and depending on net usage rates, subsequent hang-up campaigns will be organized to increase net usage. Throughout the study, community health workers will be utilized to encourage continuous net use in the study population. Hamlet and religious leaders will be also involved in the sensitization campaigns for net usage.

The coverage achieved in each cluster will be evaluated through a post-intervention coverage survey one month after distribution. Net coverage and usage will also be assessed during cohort visits and cross-sectional surveys. Three indicators will be used: “proportion of households with at least one ITN for every 2 people”, “proportion of household members with enough ITNs to sleep under (population access)” and “proportion of residents reporting using an ITN last night” [36, 37].

### Outcomes

The *primary outcome* is malaria case incidence in children aged 6 months to 10 years (Table 1) over 24 months. A malaria case is defined as an infra-rouge frontal temperature above 37.5 °C or history of a fever in the last 48 h and a positive rapid diagnostic test (RDT).

**Table 1:** Study outcomes, measurements, and process of collection

Outcome	Measurement	Collection
**Epidemiological outcomes**		
Malaria case incidence	Rapid diagnostic test taken when temperature ≥ 37.5 °C and/or history of fever for the past 48 h	Active case detection: cohort follow-up
Malaria infection prevalence	Rapid diagnostic test whatever the presence of malaria signs	Cross-sectional survey
Moderate to severe anaemia	Haemoglobin (measured by Haemocue), defined as < 10 g/dL and < 8 g/dL, respectively.	Active case detection: cohort follow-up Cross-sectional survey
Temperature	Digital infra-red ear thermometer Temperature and history of fever	Active case detection: cohort follow-up Cross-sectional survey
**Entomological outcomes**		
Indoor and outdoor *Anopheles* biting density	Human landing catch	Entomology monitoring
Mosquito sporozoite rate	Standard CSP-ELISA uses to estimate the EIR. The positive sample will be confirmed with a second ELISA using a heating technique	Entomology monitoring
*Anopheles* species identification	*An. gambiae*, *An. coluzzii* and *An. funestus* Taq Man Real time PCR	Entomology monitoring Sentinel site and resistance test
Resistance intensity	WHO cylinder assay, collection of adult *Anopheles* resting indoor	Entomology monitoring
Frequency of Vgsc mutation	Taq Man PCR	Entomology monitoring
Identification and intensity of overexpressed CYP6 genes	Screening for CYP6 genes by using Microarrays Reverse-transcription quantitative PCR (RT-qPCR) using for confirming and monitoring the expression of the cytochrome identified	Resistance activity, unfed 3 days old adult *Anopheles* from larval collection

*Abbreviations*: *EIR* Entomological inoculation rate

#### Secondary outcomes are

- Malaria infection prevalence in the study population at 6 and 18 months post bed net distribution,
- Prevalence of moderate to severe anaemia in children under 5 years old at 6 and 18 months post bed net distribution,
- EIR, as a measure for malaria transmission rate in the primary vector species.

*Other outcomes* are:

- Mosquito density and survivorship, mosquito resting behaviour, species composition, ovary development, and fecundity.
- Frequency and intensity of phenotypic and genotypic resistance to pyrethroid, chlorfenapyr, and pyriproxyfen insecticides.
- Prevalence of elevated cytochromeP450s and other metabolic enzymes associated with pyrethroid resistance [38].

### Sample size

Sample size calculation for the primary outcome (malaria case incidence in children aged between 6 months and 10 years) was based on the method of Hayes and Bennett [39]. Based on passive data collected in 2018 by the NMCP in the study area, the mean number of malaria episodes per child per year in the reference arm was assumed to be 1. We assumed a between-cluster coefficient variation of 0.3 and that dual-AI LLINs will reduce malaria case incidence by 30% (i.e. to 0.7 cases per child per year). With a follow-up of 2 years, to detect this effect size with 80% power would require following 20 children and 5 additional children to account for the loss of follow-up (25 in total) in 20 clusters in each of the three arms. This sample size calculation includes adjustment for multiple testing (allowing for the 3 arms) using a Bonferroni-corrected two-sided alpha of 1.67%. This results in a total of 60 clusters required for the trial and a cohort of 1500 children.

For the cross-sectional surveys, it was assumed that malaria prevalence in the reference arm is 40%, with a coefficient of variation between clusters of 0.3. With 70 individuals per cluster and 20 clusters per arm, the study will have 80% power to detect a relative 30% lower prevalence (prevalence ratio 0.70) between each intervention arm to standard LLIN using a Bonferroni-corrected two-sided alpha of 1.67% to account for multiple comparisons.

To achieve adequate participant enrolment to reach the target sample size, enhanced community sensitization activities will be conducted before and during each activity in the community with the support of hamlet leaders and community health workers.

### Assignment of interventions: allocation and blinding

Restricted randomisation will be used to allocate the 60 clusters into the three study arms. Arms will be balanced on the following criteria: population size of the cluster (required to balance net numbers), infection prevalence, and socio-economic status. Data on these variables by cluster will be obtained through the pre-trial (baseline) cross-sectional survey.

The study is a single-blinded trial. Study participants will be blinded to the type of nets they have received. All field staff will be blinded to the allocation and analyses will be conducted on blinded data.

### Data collection, management, and analysis

#### Census

A short questionnaire will be used to collect demographic details on household residents to estimate the number of nets required to be distributed in each house and to randomly select children for the cohort. Each building will be mapped using a Global Positioning System to assist with delineating clusters.

#### Cohort monitoring

All children included in the cohort will be cleared of infection at enrolment using directly observed treatment with artemisinin-based combination therapy (ACT). During follow-up, visits will take place every two weeks during the malaria transmission season (from April to November) and every month during the dry season (from December to March). During each visit, children with fever (temperature ≥ 37.5 °C) or reporting a fever in the past 48 h will be tested for the presence of malaria parasites using RDT (SD Bioline Malaria Ag P.f, HRP2, Standard Diagnostics, Germany). If a child has a positive RDT, they will be recorded as a malaria case and will receive treatment as per national guidelines [40].

During the enrolment visit, a detailed questionnaire will be administered, to collect information on net ownership, household assets (as a proxy for socioeconomic status) and housing materials and window screening, and a clinical examination of the child will be conducted. In follow-up visits, a short questionnaire will be administered to record information on LLIN usage the night before, any adverse events encountered, and travel history. At each visit, children will also be examined by study nurses for signs of other illnesses and will be treated if needed. A child will be considered “lost to follow-up” if they miss at least 4 consecutive visits (i.e. 8 weeks of follow-up time).

#### Cross-sectional surveys

Each survey will include two components; i) a household survey and ii) a clinical survey. During the household survey, a questionnaire will be administered to obtain information on demographic and socioeconomic data, educational status, wealth assets of the household, vector control measures, and randomly sampled persons (2 per household) will be tested for malaria using an RDT, regardless of symptoms. Haemoglobin level will be measured with an HemoCue device (HemoCue Hb 201+, Aktiebolaget Leo Diagnostics, USA) in children under 5 who will receive iron tablets if they are found to be anaemic. Information will be collected on the perception and acceptance of nets. The occurrence of adverse events will also be reported.

#### Entomology activities

Entomological monitoring will take place every 3 months in each of the 60 study clusters. A standardized questionnaire will be used in each selected household to record the number of inhabitants, type of house (wall, roof, number of rooms, number of sleeping places), presence of animals, and malaria prevention measures used by household members. Mosquito collections will take place using HLCs. Four trained volunteer mosquito collectors from each study cluster will participate in house collections; one will be seated indoors and the second outdoors, and both will be replaced after 6 h of collection by the other two collectors. The collectors will be supervised by a team leader during the collection night.

Mosquitoes collected will be identified by microscopy and the numbers of *An. gambiae* s. l., *An. funestus* s. l. and other anophelines recorded [41]. Human biting rate (outdoor and indoor) will be compared between study arms. Parity rates will be estimated in a subset of live *Anopheles* mosquitoes through dissection of ovaries [42]. The presence of *Plasmodium falciparum* circumsporozoite protein (Pf-CSP) in *Anopheles* vectors will be identified in a sub-sample (minimum of 50 *Anopheles* from indoor and outdoor collections per house) using an enzyme-linked immunosorbent assay (ELISA) [43]. All *An. gambiae* s.l. positive for Pf-CSP and a sub-sample of negative specimens will be identified to species-level by PCR [44]. The EIR will be calculated as the aggregated mean of sporozoite infective bites/person/year. In order to assess the impact of the pyrethroid-pyriproxyfen LLIN (Royal Guard®) on mosquito reproduction, blood-fed *Anopheles* will be collected and ovary development recorded each year in houses from study arms, by dissection.

Insecticide resistance will be monitored annually using standard CDC intensity bottle assays [45] in the three arms to determine the prevalence of any selection of resistance to pyriproxyfen or chlorfenapyr, or change in resistance to pyrethroids. To monitor changes in insecticide resistance, adult *Anopheles* will be tested in CDC bottle bioassays using discriminating dosages of alpha-cypermethrin (12.5 μg/ml), chlorfenapyr (100 μg/ml) and pyriproxyfen (100 μg/ml) [46]; where mosquito mortality is < 90% to alpha-cypermethrin, intensity assays, testing two, five and ten times the discriminating dose will be performed. The percentage mortality at 30 min and 24, 48, and 72 h post-exposure will be recorded. Mosquitoes exposed to pyriproxyfen will be dissected to assess ovary development [47]. Tests will be performed at baseline and once per year post-intervention in two clusters per study arm.

All knockdown/dead mosquitoes after insecticidal exposure and surviving 72 h post-exposure to insecticides will be stored individually in RNAlater® and preserved at − 20 °C for gene expression analysis. Following species identification by PCR, RNA will be extracted from pools of confirmed *An. gambiae* s.s. or *An. coluzzii* and cDNA synthesized, according to standard procedures. Relative expression of CYPs and other metabolic enzymes, previously identified as being over-expressed in resistant *An. gambiae* s.l. will be measured using multiplex TaqMan RT-qPCR assays [48].

### Data management

Household data, clinical measurements in the cohort study, and entomological data collected during the cross-sectional surveys will be captured in electronic forms on smartphones installed with Open Data Kit (ODK) collect. The data will be stored on a secure server located at the London School of Hygiene and Tropical Medicine (LSHTM) and all data management and analyses will be done using Stata software.

#### Data quality and control (QC)

Standard operating procedures (SOPs) for data collection will be developed and field study staff will be appropriately trained to ensure rigorous data collection. QC will be conducted by a supervisor who will monitor the performance of field staff by checking for completeness and internal consistency of responses on the same day as data collection. Data collected on paper forms (mosquito identification, insecticide resistance results) will be double entered into a database independently by two data clerks. During the study, the data manager will prepare regular QC reports to document the status of data entry and any corrective actions needed.

##### Data security

All data will be uploaded into a secure server on the LSHTM cloud. Data will be stored encrypted and will be accessible only by secure encryption keys and passwords. Access to the data will be restricted only to authorised study investigators and data management staff. A unique identifier number will be given to each participant and household to safeguard confidentiality.

##### Data storage

Upon completion of the study, electronic files will be stored on a server and also copied to encrypted USB and stored offsite in a safe box. Case report forms (CRFs) will be stored in the secure archive equipped with locked cabinets for long-term storage. Electronic data and paper source records will be retained for a minimum of 10 years following the study completion.

### Statistical methods

Descriptive statistics will be used to compare the characteristics of participants between the study arms. All primary analyses will be conducted by intention to treat and adjusted for the restriction variables used for the constrained randomisation.

#### Primary outcome

To assess whether the new dual-AI LLINs are superior to the reference LLIN, malaria case incidence in each intervention arm (Royal Guard and Interceptor G2) will be compared to malaria case incidence in the reference arm (Interceptor). To adjust for the increased risk of type I error due to multiple pairwise comparisons, the level of significance will be adjusted using the Bonferroni method. Following any treatment for malaria, a child will not be considered at risk for two weeks. Multilevel mixed models with random effects will be used to test the difference in incidence rate between each intervention and the control arm, allowing for repeated measurements on the same individual, and within-cluster correlation of responses. Survival analyses will be also used to compare time to the first infection case, adjusting for confounding factors using a Cox proportional hazards model. Multiple events will be allowed per child (with appropriate censoring to account for prophylactic protection following treatment and absence during follow-up visits).

#### Secondary outcomes

Prevalence of malaria infection in the study clusters will be estimated at baseline, 6 months, and 18 months. The surveys will be analysed separately and in a combined model, controlling for the time in the analyses. All analyses will account for the multilevel nature of the data using random-effects regression models. The prevalence of moderate and severe anaemia in children under 5 will be analysed using logistic regression.

EIR will be estimated as the mean number of sporozoite infected *Anopheles* (*An. gambiae* s.I.) collected in the HLCs per house per night and weighted to account for the proportion of collected *Anopheles* processed for sporozoites. Differences in *Anopheles* density and EIR between the different arms will be estimated using random-effects negative binomial regression taking into account the intra-cluster correlation and any confounding factors that are found to be imbalanced between arms. Random effects logistic regression will be used to compare sporozoite rates between study arms allowing for within cluster correlation of responses.

### Oversight and monitoring

A Trial Steering Committee (TSC) and Data Safety and Monitoring Committee (DSMC) will be established to provide oversight for the study. The TSC and DSMC members will be independent of the trial and its institutions and have the necessary expertise to monitor study progress and participant safety. The DSMC will be responsible for monitoring the progress of the trial, adherence to the protocol, the safety data, and the critical efficacy endpoints.

The management of the trial is the responsibility of the chief investigator and co-investigators. They will ensure that data are recorded in compliance with good clinical practice, and all regulatory and institutional requirements.

Although the risk to study participants is considered minimal from any of the study LLINs, their safety profile will be documented. Any adverse events (AE) which have been reported to be associated with exposure to insecticides such as skin rashes, skin burning, skin itching, skin paraesthesia, watering eyes, runny nose, sneezing, mucosal irritation, headache dizziness, and their severity will be actively and passively monitored in the study cohort and study participants selected during the cross-sectional survey. Serious adverse events (SAE) will be reported to DSMC and ethical committees. Field workers will be trained to handle any disclosure of personal information or health conditions reported sensitively and confidentially.

### Ethics and dissemination

#### Research ethics approval

This study has been approved by the ethical review committee of the Ministry of Health in Benin (N°6/30/MS/DC/DRFMT/CNERS/SA), the institutional review board of London School of Hygiene and Tropical Medicine (N°16,237), and the WHO Research Ethics Review Committee (ERC.0003153). Any amendments to the protocol and informed consent forms will be submitted for approval to all three ethics committees. The trial is registered on clinicaltrials.gov (NCT03931473, registered on 30 April 2019).

The study will be conducted according to the Declaration of Helsinki and the International Guidelines for Ethical Review of Epidemiological Studies [49]. All field and clinical staff as well as the investigators will receive training on good clinical and laboratory practice before data collection starts.

#### Informed consent procedure

Before any project activities, villages and hamlet leaders and local health staff will be invited to sensitisation sessions. Community health workers within each cluster will be fully informed as to the aims of the trial and will be on hand to answer day to day questions regarding the study.

For all activities involving a household, written informed consent will be obtained from an adult guardian in the household or be given by the participant if over 18 years. The consent form will be in French and indicate the purpose of the study, procedures, risks, and benefits. All participation is completely voluntary, and participants can withdraw at any time. In case the participant does not understand French, study investigators will be trained to practice oral translation of the consent into local languages. Participants will be asked to sign the consent in duplicate, one will be kept by the project and the other will remain with the household. If the person consenting is unable to read or write, their fingerprint will be taken, and an impartial witness to the informed consent procedures signature will be requested to sign. For the active follow-up cohort, consent will be sought once at the first enrolment visit and will be performed by the study nurses. Assent will be sought for children over 10 years.

For HLCs, written informed consent will also be obtained from volunteer mosquito collectors before being involved in the study. Mosquito collectors will be over the age of 18 years. To prevent yellow fever, they will be vaccinated. Their health will be monitored by the project clinical team and free treatment for malaria will be provided, if necessary. Each volunteers will work one night every 3 months to reduce the risk.

#### Confidentiality

All procedures for data collection, management, storage, and manipulation will follow SOPs. To ensure confidentiality is maintained, paper CRFs with participants’ names will be stored in a locked cabinet and only accessible by authorised staff. All analyses will be done using the unique identifier.

#### Dissemination activities

All findings of the trial will be shared with international policymakers such as the WHO Vector Control Advisory Group (VCAG), the malaria policy advisory group (MPAC), and the WHO prequalification team for vector control product for revision of WHO guidance on dual-AI LLINs. The project will involve the engagement of national, local, and community authorities and leaders. Mid- and end of project meetings will be organized to share the progress with village and community leaders to help disseminate findings to the community.

## Discussion

A new generation of ITNs is urgently needed to maintain and ideally improve on the gains achieved by standard pyrethroid LLINs over the past two decades [18] and to control malaria transmitted by pyrethroid-resistant vectors. There is still only minimal evidence for the use of these nets in areas of insecticide resistance [9, 10, 12], and more evidence is required for consolidating the findings of these initial trials.

The most advanced dual-AI LLIN assessed until now are nets combining a synergist PBO and pyrethroid insecticide. Two large RCTs conducted in Tanzania [9] and Uganda [10] have shown better efficacy than standard LLINs on malaria infection prevalence. This has led to a recommendation from WHO that PBO nets can be deployed across SSA in areas where vectors are resistant to pyrethroid insecticides [50].

Pyriproxyfen-pyrethroids LLINs have shown improved efficacy and wash resistance relative to pyrethroid-treated nets in terms of mosquito mortality and prevention of blood-feeding in experimental hut trials in Benin [11]. Several phase II studies conducted in West Africa have also shown that this type of dual-AI LLIN had a significant impact on mosquitoes’ fertility by sterilising a large proportion of surviving blood-fed female mosquitoes through the pyriproxyfen component [13, 14, 51]. To date, there is only one RCT that assessed pyriproxyfen-pyrethroid LLINs (Olyset® Duo). The trial was conducted in Burkina Faso over 18 months and showed 12% protective efficacy compared to the standard net on malaria case incidence but no reduction in infection prevalence [12]. The relatively small protective effect could be due to the short residual bio-efficacy of pyriproxyfen on the net. Indeed, the study investigators reported that fecundity indicators were only reduced in the first month after the use of the pyriproxyfen-pyrethroid LLINs, but not thereafter [52]. Royal Guard®, also a dual pyriproxyfen-pyrethroid LLIN, [14] induced greater levels of suppression of mosquito reproduction in Phase I and II trials than that which was reported in similar studies with Olyset® Duo [11]. Chlorfenapyr-pyrethroid LLINs have demonstrated better efficacy and wash resistance against pyrethroid-resistant mosquitos in comparison to standard alpha-cypermethrin LLINs in Phase I and II trials [15–17].

Currently, there is less evidence for the performance of dual-AI LLINs on epidemiological outcomes in West African communities where vectors usually display much higher levels of insecticide resistance intensity than those observed in East Africa, raising the concern that they might not be as effective in areas with intense insecticide resistance. The present trial will therefore answer a critical question about the efficacy of the most promising generation of LLINs in West Africa (except for PBO-pyrethroid LLINs which were not included). It is the only clinical trial in the area to assess mixtures of adulticides with different modes of action (chlorfenapyr and pyriproxyfen) with alpha-cypermethrin. The findings of this trial together with those of a similar RCT conducted in Tanzania will be reviewed by the WHO VCAG and will contribute to the body of evidence to enable policy recommendations. If these two first-in-class products demonstrate superior efficacy to standard LLINs, they will be the first recommended LLINs impregnated with an insecticide class other than pyrethroids and PBO in the last three decades and will pave the way for the development of further LLINs. Any dual-AI LLIN that will be developed subsequently and share the same mode of actions would need to show non-inferiority against entomological outcomes in Phase II experimental hut conditions to be prequalified by WHO [53].

LLINs combining two insecticides of unrelated classes may become a key component of future insecticide resistance management by decreasing selection pressure (genotypic and phenotypic) among vector populations. While resistance monitoring in the proposed study is not powered to be able to detect significant changes, the trialling side by side of dual-AI LLINs and standard LLINs may allow us to detect important trends. Furthermore, profiling of insecticide resistance in the local vector will provide valuable information on the condition of deployment of these tools.

Both nets being tested contain a pyrethroid. The main effect of pyrethroids is to enhance the barrier offered by the netting material by killing and repelling mosquitoes and reducing blood feeding. The second AI in the dual LLINs cannot provide this level of individual protection. Neither chlorfenapyr nor pyriproxyfen has repellent effects. The main impact of the partner AIs will be to kill or sterilise mosquitoes that come into contact with the insecticides, therefore reducing the overall mosquito population. For both nets, the impact of these interventions is expected to be maximal at the community level when net usage is high. It will be therefore critical to achieve high population coverage. Several enhanced community sensitization activities and hang-up campaigns will be organized before, during, and after the LLIN distribution.

Nets have been one of the mainstays of vector control for several decades, primarily due to their ease of use and effectiveness. However, as anecdotal reports of their reduced efficacy are reported [4, 5, 54], alongside evidence that new nets provide better protection, there is a danger that community willingness to use nets may wane. It is vital that confidence is maintained in these life-saving tools. If these new generations of LLINs are effective at reducing malaria, this trial will provide vital evidence to policy-makers and will enable stakeholders to consider these dual-AI LLINs as alternative solutions for malaria control in areas of insecticide resistance.

### List of protocol version

Protocol version 4.0: 30/06/2019

### Trial status

Baseline surveys are completed, and the cohort recruitment is currently running. Due to the COVID-19 pandemic and the cases confirmed in Benin, the recruitment of cohort children has been postponed from April to July 2020. Entomological collection using human landing catches were also suspended and resumed at the end of June. Laboratory work and hut trial work continued as normal. This delay has led to the loss of 3 months of data collection; therefore, the sample size has been modified accordingly. With a follow-up of 21 months, 30 children instead of 25 per cluster would be required for detecting the impact of the study nets. For mosquito collection, each cluster will be visited seven times over the 21 months follow-up period. The amendments have been submitted to the different ethics committee. All staff is using appropriate protective equipment and hand sanitation measures.

## Supplementary Information

**Additional file 1.** Information sheet and consent forms.

## Data Availability

The full protocol is available from the study principal investigator (Dr. Natacha Protopopoff; Email: natacha.protopopoff@lshtm.ac.uk) upon reasonable request.

## Abbreviations

ACT: Artemisinin-based combination therapy
AE: Adverse event
AI: Active ingredient
CRF: Case report form
DHS: Demographic health survey
DSMB: Data safety and monitoring committee
EIR: Entomological inoculation rate
GPRIM: Global Plan for Insecticide Resistance Management in malaria vectors
HLC: Human landing catches
IEC: Information, education and communication
ITN: Insecticide-treated net
LLIN: Long-lasting insecticidal net
LSHTM: London school of hygiene and tropical medicine
MPAC: Malaria policy advisory group
MFO: Mixed function oxidases
NMCP: National malaria control programme
ODK: Open data kit
PCR: Polymerase chain reaction
PBO: Piperonyl butoxide
Pf-CSP: *Plasmodium falciparum* circumsporozoite protein
QC: Quality control
RCT: Randomized clinical trial
RDT: Rapid diagnostic test
SAE: Serious adverse event
SOP: Standard operating procedure
SPIRIT: Standard protocol items recommendations for interventional trials
SSA: Sub-Saharan Africa
TSC: Trial steering committee
VCAG: Vector Control Advisory Group
WHO: World health organization
