# Supplementary material for: Assessing the efficacy of two dual-active ingredients long-lasting insecticidal nets for the control of malaria transmitted by pyrethroid-resistant vectors in Benin: study protocol for a three-arm, single-blinded, parallel, cluster-randomized controlled trial
Source: BMC Infect Dis. 2021 Feb 19;21:194. doi: 10.1186/s12879-021-05879-1 (PMC7892705; doi:10.1186/s12879-021-05879-1)
Supplement: Supplementary file 1 — Additional file 1. Information sheet and consent forms. [file 12879_2021_5879_MOESM1_ESM.pdf]

ANNEXE: Consent forms

1 Consent form: census/mapping

INFORMATION AND CONSENT FORMS

ENUMERATION CONSENT AGREEMENT FOR VILLAGE LEADER

Introduction

Good morning. My name is __________. I work with Centre de Recherche Entomologique de Cotonou (CREC). We work together with the Zou department Health office and Cove, Zagnanado and Ouinhi district office, the National malaria control program and London School of Hygiene and Tropical Medicine. I am here to ask you some question to get some information on your house. We have two new types of insecticide treated nets and we want to find out which one works best.

Purpose

The enumeration will provide a house and household register (each with an identification code) for selected villages within Missungwi district.

What will happen

If you agree to participate, we will carry out the following activities:

1. We will create a map of your village and hamlet boundaries using a satellite-tracking device to identify the location of your house within the village.
2. We will record the names of the head and member of household as well as age and gender

Risks: We can see little risk in participating in the enumeration study.

Benefits: There will be a separate round of consent for the intervention study. The results of the study will help us learn how best malaria can be controlled.

Confidentiality: Your individual information will be kept private. The information gathered will be for use in implementation and monitoring of the trial only and shall not be disclosed to any external or third party.

Institutional Approval: This study has been approved by the Ethics Committee of the London School of Hygiene and Tropical Medicine, of the Comité Nationale d'Ethique pour la Recherche en Santé (CNERS) and of the World health Organisation.

Costs and compensation for participating in the study

You will not be asked to pay anything for you to participate in this study. The study will not reimburse you with any payment for taking part in the study.

The London School of Hygiene and Tropical Medicine is the Sponsor and hold insurance policies which apply to this study

If you have any questions or clarification pertaining to this project, please feel free to ask the field assistants or you may contact Study scientist; Dr. Corine Ngufor (+229 67164499) of CREC/LSHTM and Prof. Martin Akogbeto (+229 97012545) of CREC.

If you have any questions about your rights as a study patient, or if you think your child has been injured because of this study, please contact the Chairman of the Comité Nationale d'Ethique pour la Recherche en Santé (CNERS) on +22921332178/63

2 Consent forms: Household and prevalence survey

HOUSEHOLD AND MALARIA PREVALENCE SURVEY INFORMED CONSENT AGREEMENT

Introduction

Good morning. My name is __________. I work with Centre de Recherche Entomologique de Cotonou (CREC). We work together with the Zou department Health office and Cove, Zagnanado and Ouinhi district office, the National malaria control program and London School of Hygiene and Tropical Medicine. I am here to ask you some question to get some information on your house. We have two new types of insecticide treated nets and we want to find out which one works best. To do this, we are gathering information by visiting a number of households in your community.

Purpose of the survey

To see if the malaria program works, we would like to ask you some general questions about your household, bed-net possession, and use. We want to see how common malaria is among people in your community by testing for parasites in their blood. Your responses to our questions and the results of our studies will help us learn how best to further improve malaria control in your community and in the country.

Procedures

- If you agree to take part, we will ask you a number of questions about your family and household about bed nets used. Some people will be selected to be tested for malaria. I will ask all selected people to go to see a CREC-employed nurse on the ____________ (give the date of the consultation) in _____________ (give the place). The nurse will take several small drops of blood from each selected person. The whole process should take about 30 minutes.
- The nurse will take a small amount of blood from the finger using a small needle. One drop of blood will be used to test for a rapid malaria diagnostic test, and other drop to prepare the blood slide. This blood slide will be analysed in a laboratory in CREC and may need to be kept for further analysis after the survey. A drop of blood will also be used to test for anaemia. The identity of the person will not be connected to these samples. We will also test whether the person currently has fever.
- The results from the malaria rapid diagnostic test will be given the same day. If the person has malaria or fever, he will be provided with free drugs by the CREC clinician. In case the person does not get better, you are requested to go to the nearest health facility immediately to receive alternative treatment according to the Ministry of Health policies. If we diagnose any person as having severe malaria or other diseases you will be immediately referred to nearby health facilities.
- Net inspection: If you agree to take part in this survey, we will ask you additional questions about the net you have (washing, type of sleeping bed and repair) and would like also to see and inspect nets in your house to assess the quality. This will help us determine for how long these nets can sustain different field condition. Therefore, enable us devise alternative measures for improving and strengthening nets to meet community needs. I will not damage the net, and after the interview and will return it after the inspection.

Risks

The tested person will feel pain for a few seconds when we take the blood from his/her finger. The malaria drugs given are proven to be safe and effective, but any drugs can cause side effects in a small number of patients. The nurse will discuss if treatment is needed.

Benefits

If the malaria test shows that your child has malaria, or fever at the time of the survey, they will receive free treatment that the Ministry of Health recommends.

Voluntariness and confidentiality

It is entirely your choice to take part in or not take part in this survey as I have just described it. If you do agree to take part, your individual answers to all questions and the test results will be kept private and not revealed to anyone. If you agree to take part, you can also decide not to answer any of the questions that you do not want to, and you can refuse the blood tests.

Costs and compensation for participating in the study

You will not be asked to pay anything for you to participate in this study. The study will not reimburse you with any payment for taking part in the study.

Consent for long-term sample storage for future studies

We are also asking people who join this study if they will let the researchers' use their blood sample for future studies. These future studies may help find new ways to prevent malaria or other diseases. If you agree, we will store your blood in the laboratory with a unique number and not with your name. Your sample will be stored for up to 25 years. We may share your test results with researchers at other organizations, but we will not give them your name, address, or any information that could identify you. After the study has ended, we will remove any means to link the sample to you, and we will not be able to find your sample. If you do not wish to have your blood stored for future tests, you may still participate in our study.

The London School of Hygiene and Tropical Medicine is the Sponsor and hold insurance policies which apply to this study

Thank you very much for your time. Would you like to take part in this survey?

HOUSEHOLD COPY – Household and child – Date: ___/___/___

Consent

- The study has been explained to me, I have been given the opportunity to ask questions concerning this study. Any such questions have been answered to my full satisfaction. I understand participation is voluntary and I may revoke this consent at any time without penalty or loss of benefits, if any.
- I agree for me and my child/children to take part.
- I agree that the data generated from this study can be used in the future for other malaria related research. Yes |___| No |___|
  I agree that the dried blood samples stored can be used in the future for other malaria related research. Yes |___| No |___|

Name of participant.. Signature/Thumb print ....

Relationship to the children…………………………………………….…..

Name of the witness..Signature……………………………………………………......

Name of interviewer…………………………………….............Signature..

If you have any questions or clarification pertaining to this survey please feel free to ask the field workers and nurse or you may contact Dr. Corine Ngufor (+229 67164499) of CREC/LSHTM, Prof. Aurore Hounto (+229 97684645) of NMCP and Prof. Martin Akogbeto (+229 97012545) of CREC.

If you have any questions about your rights as a study patient, or if you think your child has been injured because of this study, please contact the Chairman of the Comité Nationale d'Ethique pour la Recherche en Santé (CNERS) on +22921332178/63

PROJECT COPY – Household and child

Date: ___/____/_____ Cluster Number: ___ ___ Household Number: ___ ___ ___

MALARIA PREVALENCE SURVEY INFORMED CONSENT AGREEMENT FOR THE PARENT / GUARDIAN OF CHILDREN IN THE TRIAL

Consent

- The study has been explained to me, I have been given the opportunity to ask questions concerning this study. Any such questions have been answered to my full satisfaction. I understand participation is voluntary and I may revoke this consent at any time without penalty or loss of benefits, if any.
- I agree for me and my child/children to take part.
- I agree that the data generated from this study can be used in the future for other malaria related research. Yes |___| No |___|
  I agree that the dried blood samples stored can be used in the future for other malaria related research. Yes |___| No |___|

Name of participant.. Signature/Thumb print ....

Relationship to the children……………………………………………….

Name of the witness..Signature…………………………………………………....

Name of interviewer………………………..........................Signature...

If you have any questions or clarification pertaining to this survey please feel free to ask the field workers and nurse or you may contact Dr. Corine Ngufor (+229 67164499) of CREC/LSHTM, Prof. Aurore Hounto (+229 97684645) of NMCP and Prof. Martin Akogbeto (+229 97012545) of CREC.

If you have any questions about your rights as a study patient, or if you think your child has been injured because of this study, please contact the Chairman of the Comité Nationale d'Ethique pour la Recherche en Santé (CNERS) on +22921332178/63
on +22921332178/63

3 Consent forms: Children cohort follow up

CHILDREN COHORT FOLLOW UP CONSENT FORM

Introduction

Good morning. My name is __________. I work with Centre de Recherche Entomologique de Cotonou (CREC). We work together with the Zou department Health office and Cove, Zagnanado and Ouinhi district office, the National malaria control program and London School of Hygiene and Tropical Medicine. I am here to ask you some question to get some information on your house. We have two new types of insecticide treated nets and we want to find out which one works best.

Purpose of the survey

To see if the mosquito net distributed works. We want to know if, children in your community have malaria by taking their temperature and testing for parasites in their blood if they feel unwell. Your responses to our questions and the results of our studies will help us learn how best to further improve malaria control in your community and in the country.

Procedures

If you agree to take part, we will select at random one child from 6 months to 10 years from your household to attend every two weeks the CREC mobile clinic located in your village. We will ask you some questions about the child, including if he had fever, if he is sick and received a treatment, bed-net use and any side effect from the use of bed net. We will take his temperature. If your child has symptoms of malaria infection the nurse will take several small drops of blood to diagnose malaria. The whole process should take about 30 minutes. The selected child will be followed for one year or until he/she reach 10 years old.

To diagnose malaria, the nurse will take a small amount of blood from the finger using a small needle. One drop of blood will be used to test for a rapid malaria diagnostic test and other drop to prepare the blood slide. This blood slide will be analysed in a laboratory in CREC and may need to be kept for further analysis after the survey. The identity of the child will not be connected to these samples.

The results from the malaria rapid diagnostic test will be given the same day. If the person has malaria or fever, he will be provided with free drugs by the CREC clinician. In case the person does not get better, you are requested to go to the nearest health facility immediately to receive alternative treatment according to the Ministry of Health policies. If we diagnose any person with severe malaria or other diseases, you will be immediately referred to nearby health facilities for free.

Risks and Benefits

The tested person will feel pain for a few seconds when we take the blood from his/her finger. If the test shows that your child has malaria, or fever at the time of the survey, they will receive free treatment that the Ministry of Health recommends. These drugs are proven to be safe and effective, but any drugs can cause side effects in a small number of patients. The nurse will discuss if treatment is needed.

Voluntariness and confidentiality

It is entirely your choice to take part or not in this survey as I have just described it. If you do agree to take part, your individual answers to all questions and the test results will be kept private and not revealed to anyone. If you agree to take part, you can decide not to answer some of the questions, and you can also refuse the blood tests.

Costs and compensation for being in the study

You will not be asked to pay anything for you to participate in this study. You will receive reimbursement for your transport to come to the mobile clinic and go back home. The total amount will be on average 500 FCFA for each visit.

Consent for long-term sample storage for future studies

We are also asking people who join this study if they will let the researchers' use their blood sample for future studies. These future studies may help find new ways to prevent malaria or other diseases. If you agree, we will store your blood in the laboratory with a unique number and not with your name. Your sample will be stored for up to 25 years. We may share your test results with researchers at other organizations but we will not give them your name, address, or any information that could identify you. After the study has ended, we will remove any means to link the sample to you, and we will not be able to find your sample. If you do not wish to have your blood stored for future tests, you may still participate in our study.

The London School of Hygiene and Tropical Medicine is the Sponsor and hold insurance policies which apply to this study

Thank you very much for your time. Would you like to take part in this survey?

HOUSEHOLD COPY – Household and child – Date: ___/___/___

CONSENT

- The study has been explained to me, I have been given the opportunity to ask questions concerning this study. Any such questions have been answered to my full satisfaction. I understand participation is voluntary and I may revoke this consent at any time without penalty or loss of benefits, if any.
- I agree for me and my child/children to take part.
- I agree that the data generated from this study can be used in the future for other malaria related research. Yes |___| No |___|
  I agree that the dried blood samples stored can be used in the future for other malaria related research. Yes |___| No |___|

Name of guardian/parent.. Signature/Thumb print ....

Name of the child selected……………………………………Relationship to the chil…………………………………………

Name of the witness...Signature…………………………………………………….....

Name of interviewer……………………………………………….....Signature..

If you have any questions or clarification pertaining to this survey please feel free to ask the field workers and nurse or you may contact Dr. Corine Ngufor (+229 67164499) of CREC/LSHTM, Prof. Aurore Hounto (+229 97684645) of NMCP and Prof. Martin Akogbeto (+229 97012545) of CREC

If you have any questions about your rights as a study patient, or if you think your child has been injured because of this study, please contact the Chairman of the Comité Nationale d'Ethique pour la Recherche en Santé (CNERS) on +22921332178/63

PROJECT COPY – Household and child

Date: ___/____/_____ Cluster Number: ___ ___ Household Number: ___ ___ ___

CHILDREN COHORT FOLLOW UP CONSENT FORM

CONSENT

- The study has been explained to me, I have been given the opportunity to ask questions concerning this study. Any such questions have been answered to my full satisfaction. I understand participation is voluntary and I may revoke this consent at any time without penalty or loss of benefits, if any.
- I agree for me and my child/children to take part.
- I agree that the data generated from this study can be used in the future for other malaria related research. Yes |___| No |___|

I agree that the dried blood samples stored can be used in the future for other malaria related research. Yes |___| No |___|

Name of guardian/parent... Signature/Thumb print ....

Name of the child selected……………………………………Relationship to the child…………………………………………

Name of the witness..Signature…………………………………………………………….

Name of interviewer………………………………………………..Signature...

If you have any questions or clarification pertaining to this survey please feel free to ask the field workers and nurse or you may contact Study staff; Dr. Corine Ngufor (+229 67164499) of CREC/LSHTM, Prof. Aurore Hounto (+229 97684645) of NMCP and Prof. Martin Akogbeto (+229 97012545) of CREC;

If you have any questions about your rights as a study patient, or if you think your child has been injured because of this study, please contact the Chairman of the Comité Nationale d'Ethique pour la Recherche en Santé (CNERS) on +22921332178/63

4 Consent forms: Mosquito trapping

Mosquito trapping informed consent agreement

Introduction

Good morning. My name is __________. I work with Centre de Recherche Entomologique de Cotonou (CREC). . We work together with the Zou department Health office and Cove, Zagnanado and Ouinhi district office, the National malaria control program and London School of Hygiene and Tropical Medicine. I am here to ask you some question to get some information on your house. We have two new types of insecticide treated nets and we want to find out which one works best.

Purpose of the survey

We would like to include you in a study to find out if different new nets products as one of the control intervention can reduce the transmission of malaria in your communities. Malaria is transmitted by mosquitoes that carry the malaria parasite. The control interventions reduce the number of infected mosquitoes. We want to find out whether the LLINs reduce the number of mosquitoes flying into your house. It will provide information on which new LLINs works best to reduce mosquito numbers and malaria.

Procedure for light trap catching

If you agree to participate, we will carry out the following activities:

1. Collection of mosquitoes using a special light trap in your bedroom for one night. The trap will collect mosquitoes coming indoors and we will collect the trap early the following morning. The trap light will be turned on in the early evening and will be on through the night. You and others in the room will sleep under a bed net which we will provide for you, and for the others *if necessary*, on the night we collect mosquitoes.
2. You will be asked to complete a short questionnaire. We will ask a few questions about your house and any mosquito control you may have used. For this process you will be identified by a study code, not by your name, so that the views you express and answers you provide will remain completely anonymous.

Procedure for Human Landing catches catching

If you agree to participate, we will carry out the following activities:

1. Collection of mosquitoes using human landing catches. We will ask if you will allow volunteers to collect mosquitoes inside and outside your house. They will sit during the night in one of your room and also outside near by your house from 7 pm to 7 am.
2. You will be asked to complete a short questionnaire. We will ask a few questions about your house and any mosquito control you may have used. For this process you will be identified by a study code, not by your name, so that the views you express and answers you provide will remain completely anonymous.

Procedure for tent trap collection

If you agree to participate, we will carry out the following activities:

1. Collection of mosquitoes using a special trap that will be set up outside your house. The trap will collect outdoors mosquitoes. We will ask you permission to install a tent nearby your houses and allow us to sleep under.
2. You will be asked to complete a short questionnaire. We will ask a few questions about your house and any mosquito control you may have used. For this process you will be identified by a study code, not by your name, so that the views you express and answers you provide will remain completely anonymous.

Procedure for collection of resting mosquitoes

1. We will collect mosquitoes resting on your wall and inside your net early on the morning around 6-7 am.
2. For this process your house will be identified by a study code, so that the result cannot be related to you

Voluntariness and confidentiality

It is entirely your choice to take part in or not take part in this survey as I have just described it. If you agree to take part, you can also decide not to answer any of the questions that you do not want to. You can also decide at any moment during the night collection to stop the trap or ask the volunteers to leave. We will collect the material on the morning. Your individual information will be kept private.

Risks and Benefits:

We can see little risk in taking part in this study. You might hear some noise coming from the trap during the night when installed near your bed and volunteers working inside and outside the house. The volunteers have been trained to reduce the discomfort to have them in your house at minimum. If you are not sleeping under a long-lasting net you will receive one to sleep under the night the trap is running. The traps may reduce the number of mosquitoes in your house. The results of the study will help us learn how best malaria can be controlled.

Costs and compensation for participating in the study

You will not be asked to pay anything for you to participate in this study. The study will not reimburse you with any payment for taking part in the study.

The London School of Hygiene and Tropical Medicine is the Sponsor and hold insurance policies which apply to this study

Thank you very much for your time. Would you like to take part in this survey?

HOUSEHOLD COPY Date: _____/______/________

Mosquito trapping Informed Consent agreement

Consent section

- The study has been explained to me, I have been given the opportunity to ask questions concerning this study. Any such questions have been answered to my full satisfaction. I understand participation is voluntary and I may revoke this consent at any time without penalty or loss of benefits, if any.
- I agree to take part to the survey
- I agree that the data generated from this study can be used in the future for other malaria related research. Yes |___| No |___|

Name of guardian/parent.. Signature/Thumb print ....

Name of the witness...Signature...

Name of interviewer...Signature...

If you have any questions or clarification pertaining to this survey please feel free to ask the field workers or you may contact Dr. Corine Ngufor (+229 67164499) of CREC/LSHTM, Prof. Aurore Hounto (+229 97684645) of NMCP and Prof. Martin Akogbeto (+229 97012545) of CREC

If you have any questions about your rights as a study patient, or if you think your child has been injured because of this study, please contact the Chairman of the Comité Nationale d'Ethique pour la Recherche en Santé (CNERS) on +22921332178/63

PROJECT COPY   Date _____/______/______

Cluster Number: ___ ___Household Number: ___ ___ ___ Round: __ __

Mosquito trapping Informed Consent agreement

Consent section

The study has been explained to me, I have been given the opportunity to ask questions concerning this study. Any such questions have been answered to my full satisfaction. I understand participation is voluntary and I may revoke this consent at any time without penalty or loss of benefits, if any.

- I agree to take part to the survey
- I agree that the data generated from this study can be used in the future for other malaria related research. Yes |___| No |___|

I agree to take part.

Name of guardian/parent.. Signature/Thumb print ....

Name of the witness..Signature...

Name of interviewer..Signature...

If you have any questions or clarification pertaining to this survey please feel free to ask the field workers or you may contact Dr. Corine Ngufor (+229 67164499) of CREC/LSHTM, Prof. Aurore Hounto (+229 97684645) of NMCP and Prof. Martin Akogbeto (+229 97012545) of CREC

If you have any questions about your rights as a study patient, or if you think your child has been injured because of this study, please contact the Chairman of the Comité Nationale d'Ethique pour la Recherche en Santé (CNERS) on +22921332178/63
